# The role of school climate on student disclosure of private information and cyberbullying: a comparison of regular and vocational school students in China

**DOI:** 10.3389/fpubh.2024.1336617

**Published:** 2024-05-17

**Authors:** Qiqi Chen, Jiaqi Tang, Yuhong Zhu, Ko Ling Chan

**Affiliations:** ^1^Department of Applied Social Sciences, The Hong Kong Polytechnic University, Kowloon, Hong Kong SAR, China; ^2^Department of Social Work, School of Sociology and Anthropology, Xiamen University, Xiamen, China; ^3^Department of Social Work & Social Policy, Renmin University of China, Beijing, China

**Keywords:** cyberbullying, online privacy, school climate, student support, vocational school

## Abstract

**Introduction:**

Adolescents are experiencing an unprecedented cyber-saturated environment where the disclosure of private information should be approached with caution. This study aims to investigate the effects of school environment, including student support, teacher support, and opportunities for autonomy, on students’ disclosure of private information and their experiences with cyberbullying.

**Methods:**

In September 2022, a total of 1,716 students (mean age = 14.60, SD = 1.35) from three regular and vocational schools in China participated in the survey.

**Results:**

The results showed that 35.6% of the participants had experienced victimization by cyberbullying, and 12.6% had perpetrated cyberbullying. Vocational school students reported significantly higher rates of cyberbullying and lower levels of perceived school climate than students from regular school. Student support in the school environment was found to negatively affect both cyberbullying perpetration and victimization, with this impact appearing to be stronger in regular schools as compared to vocational schools. Opportunities for autonomy and the disclosure of private information were positively correlated with experiences of cyberbullying.

**Discussion:**

This study introduces a novel perspective that perceived school climate influences adolescents’ disclosure of private information and their involvement in cyberbullying. The findings could provide implications for future research and practices aimed at child protection in cyberspace.

## 1 Introduction

### 1.1 Prevalence of cyberbullying

Cyberbullying is a significant public health issue worldwide and is increasingly prevalent among children and adolescents (1). Cyberbullying is defined as repeated and deliberate aggressive behaviors carried out in cyberspace through electronic media (2). The literature indicates that the incidence rate of cyberbullying victimization can be as high as 57.5%, while rates of cyberbullying perpetration range from 6 to 46.3% (3). Recent studies have reported cyberbullying incidences of 5% in Australia, 15% in the United States, 23.8% in Canada (4, 5), 19% in Saudi Arabia, 20% in Turkey, and 37% in India (6). Although the prevalence of cyberbullying varies across countries, the number of cyberbullying perpetrators has been on the rise worldwide in recent years. An empirical study with a Chinese sample revealed that 23.5% of participants had experienced cyberbullying in the past year (7). Cyberbullying seriously impedes the physical and mental health development of adolescents, leading to a range of negative outcomes, including depression, anxiety, substance abuse, deviant behavior, and even suicide (8). Given its universality and harmful effects, cyberbullying deserves attention as a public health priority.

### 1.2 Risk factors of cyberbullying

Several individual and situational factors have been associated with cyberbullying. For example, girls are more likely to experience cyberbullying than boys due to the indirect nature of cyberbullying (9). Adolescents aged 15 years and older have a higher probability of being cyberbullying perpetrators, as older adolescents may have greater access to digital devices, be more influenced by their peers and social dynamics, and perceive themselves as more powerful or dominant compared to younger adolescents (10). Moreover, a longer duration of Internet use has been found to increase the risk of cyberbullying victimization (11). Additionally, limited emotion regulation abilities and impulsiveness are positively associated with cyberbullying perpetration (12). A systematic review identified situational factors such as negative parenting or family education, violent community environments, and poor parent–child or teacher–student relationships as risk factors for cyberbullying victimization and perpetration (3). Specifically, students who reported feelings of safety at school and supportive peer relationships were less likely to be involved in bullying or cyberbullying (13). Therefore, exploring school factors in the context of cyberbullying victimization and perpetration holds social significance.

### 1.3 School climate and adolescent adjustment

School climate is broadly defined as the quality and character of school culture and is considered a relatively stable asset for schools (14). A positive school climate has been found to be a protective factor against cyberbullying perpetration and victimization. According to ecological theories of development, autonomous self-determination and expression within schools are crucial elements for adolescent development, including academic achievement and self-esteem (15). In addition, students’ aggressive behavior is likely to escalate in the presence of deteriorating school climate, leading to an increase in cyberbullying incidents. A review of relevant literature demonstrates that teacher support, student support, and opportunities for autonomy are important components of school climate (15). Adolescents who experience positive teacher support are better equipped to navigate academic, behavioral, and emotional challenges (16). Conversely, a lack of teacher support poses a significant challenge to young people’s learning and development, potentially trapping them in the cycle of bullying (17). Positive peer influence is considered a developmental asset for adolescents, serving as a protective factor against engaging in risky behaviors (18). Opportunities for autonomy allow adolescents to make self-directed decisions regarding their studies and personal lives (19). Chinese adolescents have significantly fewer opportunities for autonomy compared to their counterparts in the United States, and the association between opportunities for autonomy and personal growth and adjustment is weaker (19).

### 1.4 Disclosure of privacy and cyberbullying

Scholars have conducted extensive research on the relationship between disclosure of private information and cyberbullying. While the Internet has brought numerous benefits to people’s lives, such as the ability to share experiences and connect with friends, it has also introduced risks such as privacy breaches and online fraud (20). Popular social networking sites often require users to provide personal information, including names, phone numbers, home addresses, and to undergo real-name authentication. With this information, social media platforms create individual profiles for users, laying the groundwork for online interactions and expanding interpersonal relationships (20). Seeking to build social networks, accumulate social capital, and be part of the trend, young people are inclined to disclose certain aspects of their private information on the Internet (21). In this process, users have the right to manage their own data, deciding and modifying the content and extent of their information disclosure to reap the benefits of Internet use. However, as the number of network users grows, a substantial amount of personal information is being disclosed and leaked, intensifying public interest in online privacy and generating widespread social concern (22).

Research has demonstrated a correlation between information disclosure and a higher incidence of cyberbullying perpetration and victimization (23). Adolescents, who may lack privacy awareness and blindly trust social platforms, are particularly vulnerable in this regard (24). Moreover, the era of big data and rapid information sharing has created more opportunities for cyberbullying to occur (25). Despite the risks, adolescents engage in online interactions, sharing personal photos and information, driven by their desire to gain popularity, even at the expense of their privacy. A recent study indicated that the availability of information on social media platforms is associated with an increase in cyberbullying incidents (26). The more private information is visible and shared widely, the greater the likelihood of experiencing cyberbullying victimization (27). Importantly, victims of cyberbullying who disclose more personal information in cyberspace not only face blamed from their environment but also receive less support and sympathy from bystanders (28).

### 1.5 Cyberbullying across school types

The research conducted in the field of cyberbullying, school climate, and disclosure of private information has been limited in examining their relationships across different types of schools. However, longitudinal studies have revealed varying rates of cyberbullying victimization across different school tracks (29). For instance, a recent study conducted in regular schools across seven provinces in China found that 23.5% of participants reported experiencing cyberbullying victimization, with 16.9% reporting being cyberbullied by their peers (30). The study also highlighted that girls were more likely to be victims of cyberbullying compared to boys. In contrast, studies focusing on Chinese vocational school students reported alarming rates of cyber-victimization at 38.8% and cyberbullying perpetration at 13.86% (31). In China, approximately 45% of students who finish their nine-year compulsory education proceed to vocational high schools, where they receive three years of training in specific trades (32). Currently, there are about 13 million vocational school students in China, a number that continues to rise (33). Vocational schools are designed to equip students with practical skills and job training across various industries, including manufacturing, technology, healthcare, and hospitality, thus preparing them for direct entry into the labor market (34). In contrast, mainstream high schools focus on cultivating students for superior academic achievements and positions in the upper echelons of society (35). Vocational school students often face greater pressures from family and job markets, and therefore may have weaker connections and support from their schools. Their sense of marginalization in society may also contribute to a higher level of distress compared to students in regular schools. Consequently, vocational school students might engage in prolonged Internet use and develop deeper connections with online acquaintances as a way to alleviate their feelings of marginalization.

Over the past two decades, China has enhanced its legal framework to protect vulnerable children, adopting nearly 20 legal measures to include comprehensive protection measures in family, school, government, judicial, and social domains (36). The 2020 revision introduced “online protection” to safeguard children from cyber threats like addiction and bullying (37). Despite these efforts, gaps remain in the systematic application of these laws, lacking a robust support system and requiring specific measures to alleviate family pressures and improve the application of legal protections (38). A multi-faceted approach involving state, schools, families, and network providers is critical to foster a supportive anti-bullying environment in schools and online platforms. Building on the existing literature, this study aimed to explore the relationship between cyberbullying, school climate, and the disclosure of private information, with a particular focus on comparing students in regular and vocational schools. The research hypotheses were as follows: (H1) a positive school climate is negatively associated with students’ engagement in cyberbullying; (H2) the disclosure of private information is positively associated with an increased risk of cyberbullying; and (H3) there are differences between regular and vocational school students in terms of cyberbullying, school climate, and the disclosure of private information.

## 2 Methods

### 2.1 Study design and procedure

In this study, participants were students in grades 8–12, aged 13–18 years, from two regular schools in Qingdao and one vocational school in Wuhan. Convenient sampling was employed with the aim of comparing cyberbullying between regular and vocational schools in China. Qingdao and Wuhan were considered new first-tier cities in China, characterized by being large, densely populated urban metropolises with significant economic, cultural, and political influence over other cities. The goal was to capture relevant dimensions of adolescent health behaviors in each city through the sample. Data collection took place during the fall semester of 2022. Approximately 2,400 students from the three schools were invited to participate, and ultimately 1,716 adolescents completed the questionnaires, resulting in a total response rate of 71.5%. Out of the respondents, 1,132 (66.0%) were students from regular schools, while 584 (34.0%) were from vocational schools. Among the participants, almost half (44.52%, *n* = 764) were male students. The mean age of the participants was 14.60 years (SD = 1.35). The majority of participants (90.03%, *n* = 1,545) had married parents at the time of the survey. Before conducting the survey, informed consent was obtained from all participating students and their parents. A trained research assistant invited the students to complete a web-based self-administered questionnaire during school hours. Measures were taken to ensure privacy by sufficiently spacing the adolescents apart from each other in the classroom. The survey took approximately 30 min to complete. The study protocol perceived approval from the Institutional Review Board of the authors’ affiliated university.

### 2.2 Measures

#### 2.2.1 Cyberbullying experiences

The participants’ experiences of cyberbullying were assessed using the Chinese version of the European Cyberbullying Intervention Project Questionnaire (ECIPQ) (3). The instrument’s reliability, validated across countries such as Spain, Germany, Italy, Poland, UK, Greece, and Colombia, showed suitable reliabilities with alphas ranging from 0.89 to 0.97 for cyber victimization and 0.83 to 0.96 for cyber aggression (39–41). The ECIPQ scale consists of 14 items, equally distributed across two subscales: cyberbullying perpetration and victimization. The CFA results revealed that the 14 items demonstrated by a *χ*^2^ of 112.87 (df = 76, *p* < 0.01), an RMSEA index of 0.047 (90% CI [0.027, 0.065]), a CFI of 0.994, a TLI of 0.992, and a WRMR of 0.578 (3). Examples of items include “I threatened someone through texts or online messages.” and “I was excluded or ignored by someone in a social networking site or Internet chat room.” Participants rated the frequency of each item on a 5-point Likert scale (0 = never, 4 = always). Mardia’s tests for skewness (*χ*^2^ = 778.58) and kurtosis (*z* = 368.69) were found to be significant at the 0.01 alpha level. The overall internal consistency of the ECIPQ scale, as measured by Cronbach’s alpha, was found to be 0.96. The perpetration and victimization subscales demonstrated high internal consistency, with Cronbach’s alpha values of 0.98 and 0.93, respectively. These values indicate strong reliability and consistency of the ECIPQ in measuring cyberbullying perpetration and victimization experiences among the participants.

#### 2.2.2 School climate

The participants’ perceived school climate was examined using a 25-item school climate measure (15). This measure evaluates three dimensions of school climate: teacher support (seven items), student–student support (13 items), and opportunities for autonomy (five items). The CFA results revealed that the scale had an acceptable fit to the data, with CFIs = 0.94, NNFIs = 0.93, RMSEAs <0.08 (14). Example items from each subscale include “The teachers believe I can do well,” “The students care about one another,” and “Students are given the chance to help make decisions.” Participants rated all items on a 4-point Likert scale (1 = never, 4 = always), while higher scores indicate greater levels of perceived support or opportunities for autonomy within the school environment. Mardia’s tests for skewness (*χ*^2^ = 159.61) and kurtosis (*z* = 28.84) were found to be significant at the 0.01 alpha level. The teacher support subscale demonstrated good internal consistency with a Cronbach’s alpha of 0.95. The student–student support subscale also showed good reliability with a Cronbach’s alpha of 0.94. The opportunities for autonomy subscale had an acceptable level of internal consistency with a Cronbach’s alpha of 0.85.

#### 2.2.3 Disclosure of private information

To assess students’ experiences of disclosing private information, a self-constructed measure consisting of 15 items was used. This measure aimed to capture the types of information disclosed, such as home addresses, odd habits, personal photos and videos, and chat histories. Each item was presented in a dichotomous format, where participants could respond with either No (coded as 0) or Yes (coded as 1) if they had experienced the disclosure of that specific type of information. In addition to assessing the types of information disclosed, participants were also asked to indicate their specific experiences related to the disclosure of private information using three options: self-disclosure (posting their own private information online), disclosure perpetration (disclosing others’ private information non-consensually), and disclosure victimization (having their private information disclosed by others without consent). Mardia’s tests for skewness (*χ*^2^ = 587.35) and kurtosis (*z* = 68.17) were found to be significant at the 0.01 alpha level. The overall Kaiser–Meyer–Olkin (KMO) value of the scale was 0.85. The total scale demonstrated good internal consistency, with a Cronbach’s alpha of 0.92. The subscales of self-disclosure, disclosure perpetration, and disclosure victimization also exhibited acceptable levels of reliability, with Cronbach’s alpha values of 0.90, 0.85, and 0.85, respectively.

### 2.3 Statistical analysis

We first computed the prevalence rates of the disclosure of private information and cyberbullying experience using descriptive statistics. Cyberbullying experiences were recoded into three clusters: perpetration, victimization, and perpetration-victimization (42). Specifically, “cyberbullying perpetration” was coded when participants responded with at least one item as 1 in the cyberbullying perpetration subscale but 0 in the cyberbullying victimization subscale. Similarly, “cyberbullying victimization” was coded when at least one item was endorsed as 1 in the cyberbullying victimization subscale but 0 in the cyberbullying perpetration subscale. Responding with at least one item as 1 in both the perpetration and victimization subscales was coded as “Perpetration-victimization.” The mean scores and standard deviations (SD) of the total scores for disclosure of private information and perceived school environment are calculated and presented. Differences in school type were tested using *t*-tests and chi-squared tests. In line with hypotheses 1 and 2, a correlation matrix was generated to explore the relationships between disclosure of private information, cyberbullying, and perceived school climate. Regression analyses were conducted to measure the effects of disclosure of private information and perceived school climate on the prediction of cyberbullying experiences, controlling for the gender and age of participants. In line with hypothesis 3, the role of school type was further examined using simple slope analysis to investigate any significant relationship between perceived school climate and cyberbullying. Statistical significance was set at *p* < 0.05, and all analyses were performed using Stata version 17.0.

## 3 Results

### 3.1 Prevalence of cyberbullying, disclosure of private information, and perceived school climate

The descriptive statistics of the participants’ reported cyberbullying experiences, disclosure of private information, and perceived school climate are summarized in Table 1. Among the participants, 35.6% reported experiencing cyberbullying victimization. Notably, 61.5% of vocational school students reported such experiences. 12.6% of the participants reported perpetrating cyberbullying, while 12.1% reported being both victims and perpetrators of cyberbullying. A significant difference was found between regular and vocational school students in terms of the prevalence of cyberbullying experiences (*p* < 0.001), with vocational school students reported a higher prevalence of all types of cyberbullying. Regarding perceived school climate, vocational school students reported significantly lower scores in all subtypes compared to their counterparts in regular schools (all *p* < 0.001), which includes lower scores in teacher support (2.76 vs. 3.11), student support (2.83 vs. 3.25), and opportunities for autonomy (2.76 vs. 2.95). Overall, the findings suggest that vocational school students have a higher prevalence of cyberbullying experiences and perceive a less supportive school climate than regular school students.

**Table 1:** Prevalence of cyberbullying, privacy disclosure, and perceived school climate.

*N* (%)	Total (*n* = 1,716)	Regular school (*n* = 1,132)	Vocational school (*n* = 584)	Chi-square/t-test
**Cyberbullying**				
Perpetration	216 (12.6)	66 (5.8)	150 (25.7)	138.02***
Victimization	610 (35.6)	251 (22.2)	359 (61.5)	259.70***
Perpetration-victimization	208 (12.1)	59 (5.2)	149 (25.5)	149.07***
**Self-disclosure (M, SD) (Posted my own information)**	1.65 (2.93)	0.99 (2.12)	2.93 (3.75)	185.57***
Name	386 (22.5)	168 (14.8)	218 (37.3)	111.75***
Birthday	398 (23.2)	162 (14.3)	236 (40.4)	147.32***
ID number	76 (4.4)	28 (2.5)	48 (8.2)	30.05***
Mobile number	253 (14.7)	88 (7.8)	165 (28.3)	128.54***
Home address	138 (8.0)	47 (4.2)	91 (15.6)	68.06***
Student card	54 (3.2)	16 (1.4)	38 (6.5)	32.79***
Sex orientation	111 (6.5)	35 (3.1)	76 (13.0)	62.68***
Sex life	30 (1.8)	9 (0.8)	21 (3.6)	17.59***
Intimate relationship	163 (9.5)	50 (4.4)	113 (19.3)	99.93***
Academic grade	315 (18.4)	159 (14.1)	156 (26.7)	41.24***
Odd habit	131 (7.6)	46 (4.1)	85 (14.6)	60.13***
Parents’ name	92 (5.4)	24 (2.1)	68 (11.6)	68.87***
Personal photos or videos	334 (19.5)	152 (13.4)	182 (31.2)	77.32***
Chat history	288 (16.8)	129 (11.4)	159 (27.2)	69.12***
Personal account password	64 (3.7)	10 (0.1)	54 (9.2)	75.05***
**Disclosure perpetration (M, SD) (Posted other’s information)**	0.48 (1.48)	0.27 (0.89)	0.90 (2.14)	80.45***
Name	141 (8.2)	62 (5.5)	79 (13.5)	33.11***
Birthday	105 (6.1)	42 (3.7)	63 (10.8)	33.59***
ID number	26 (1.5)	6 (0.5)	20 (3.4)	21.63***
Mobile number	60 (3.5)	16 (1.4)	44 (7.5)	42.77***
Home address	34 (2.0)	7 (0.6)	27 (4.6)	31.82***
Student card	15 (0.9)	3 (0.3)	12 (2.1)	14.24***
Sex orientation	30 (1.8)	12 (1.1)	18 (3.1)	9.17**
Sex life	10 (0.6)	3 (0.3)	7 (1.2)	5.80*
Intimate relationship	40 (2.3)	9 (0.8)	31 (5.3)	34.47***
Academic grade	90 (5.2)	44 (3.9)	46 (7.8)	12.34***
Odd habit	22 (1.3)	1 (0.0)	21 (3.6)	37.45***
Parents’ name	26 (1.5)	6 (0.5)	20 (3.4)	21.63***
Personal photos or videos	103 (6.0)	39 (3.4)	64 (11.0)	38.55***
Chat history	107 (6.2)	48 (4.2)	59 (10.1)	22.65***
Personal account password	18 (1.1)	2 (0.2)	16 (2.7)	24.38***
**Disclosure victimization (M, SD) (Others posted my information)**	0.47 (1.45)	0.32 (1.06)	0.75 (1.98)	44.55***
Name	156 (9.1)	77 (6.8)	79 (13.5)	21.08***
Birthday	98 (5.7)	38 (3.4)	60 (10.3)	34.23***
ID number	19 (1.1)	9 (0.8)	10 (1.7)	2.96
Mobile number	57 (3.3)	22 (1.9)	35 (6.0)	19.67***
Home address	27 (1.6)	9 (0.8)	18 (3.1)	13.01***
Student card	15 (0.9)	6 (0.5)	9 (1.5)	4.55*
Sex orientation	26 (1.5)	13 (1.2)	13 (2.2)	3.00
Sex life	12 (0.7)	6 (0.5)	6 (1.1)	1.37
Intimate relationship	38 (2.2)	14 (1.2)	24 (4.1)	14.68***
Academic grade	91 (5.3)	57 (5.0)	34 (5.8)	0.47
Odd habit	30 (1.8)	9 (0.8)	21 (3.6)	17.59***
Parents’ name	23 (1.3)	6 (0.5)	17 (2.9)	16.52***
Personal photos or videos	100 (5.8)	47 (4.2)	53 (9.1)	17.02***
Chat history	93 (5.4)	49 (4.3)	44 (7.5)	7.72**
Personal account password	14 (0.8)	2 (0.2)	12 (2.1)	16.79***
**School climate (M, SD)**				
Teacher support	2.99 (0.66)	3.11 (0.58)	2.76 (0.74)	145.57***
Student support	3.10 (0.48)	3.25 (0.44)	2.83 (0.42)	375.46***
Opportunities for autonomy	2.89 (0.71)	2.95 (0.67)	2.76 (0.75)	59.23***

*p < 0.05, **p < 0.01, and ***p < 0.001.

Regarding the disclosure of private information, vocational school students indicated significantly higher scores on all three subscales compared to regular school students. Specifically, vocational school students had higher scores in “self-disclosure” (2.93 vs. 0.99), “disclosure perpetration” (0.90 vs. 0.27), and “disclosure victimization” (0.75 vs. 0.32) (all *p* < 0.001). Among the participants, the most commonly disclosed personal information included birthdays (23.2%), names (22.5%), personal photos and videos (19.5%), and academic grades (18.4%). It is important to note that the rates of disclosure perpetration and victimization were considerably lower compared to self-disclosure rates. Regarding non-consensual disclosure of others’ information, the most frequently posted types of information without consent were names (8.2%) and chat histories (6.2%). Conversely, the types of private information most commonly disclosed by others without consent were names (9.1%) and personal photos and videos (5.8%). These findings underscore the differences in disclosure practices between vocational and regular school students. Vocational school students are more likely to disclose private information, both voluntarily and involuntarily, compared to their counterparts in regular schools, with the most commonly disclosed information including personal details such as names, birthdays, and personal media.

### 3.2 Correlations among disclosure of private information, cyberbullying, and perceived school climate

The correlations between different types of cyberbullying experiences, disclosure of private information, and perceived school climate are presented in Table 2. To examine the differences in the relationships between these measures across school types, the correlation matrix was divided. Among regular school students, all three types of perceived school climate (teacher support, student support, and opportunities for autonomy) were inversely related to all three types of cyberbullying experiences, with correlation coefficients ranging from −0.05 to −0.26 (all *p* < 0.05). In other words, higher levels of perceived school climate were associated with lower levels of cyberbullying perpetration, victimization, and dual roles of perpetration and victimization. Among vocational school students, only student support was inversely related to cyberbullying perpetration (*r* = −0.09, *p* < 0.05) and dual roles (*r* = −0.11, *p* < 0.05), while teacher support was inversely related to dual roles (*r* = −0.09, *p* < 0.05). This suggests that vocational schools, higher levels of student and teacher support were associated with lower levels of certain cyberbullying experiences. Furthermore, all three types of private information were inversely related to all three types of cyberbullying experiences for both regular (coefficients ranging from 0.13 to 0.26, all *p* < 0.001) and vocational school students (coefficients ranging from 0.13 to 0.16, all *p* < 0.05). This indicates that higher levels of private information disclosure correlate with fewer cyberbullying experiences in both school settings. However, it is important to note that no significant relationships were observed between cyberbullying perpetration or dual roles and the act of self-disclosure alone. These findings offer insights into the correlations among various factors related to cyberbullying experiences, private information disclosure, and perceived school climate, highlighting potential protective factors and distinct associations within each school type.

**Table 2:** Correlations among privacy disclosure, cyberbullying, and perceived school climate.

	Regular\Vocational	1	2	3	4	5	6	7	8	9
1	Cyberbullying perpetration	–	0.46***	0.99***	0.05	0.05	−0.25***	0.01	0.13**	0.14***
2	Cyberbullying victimization	0.41***	–	0.46***	0.07	0.07	−0.22***	0.15***	0.13**	0.16***
3	Perpetration-victimization	0.94***	0.44***	–	0.05	0.06	−0.26***	0.01	0.13**	0.15***
4	Teacher support	−0.11***	−0.14***	−0.09***	–	0.88***	0.38***	−0.09*	0.01	0.03
5	Opportunities for autonomy	−0.07*	−0.09**	−0.05*	0.74***	–	0.37***	−0.07	−0.02	0.01
6	Student support	−0.26***	−0.26***	−0.25***	0.49***	0.37***	–	−0.11*	−0.06	−0.09*
7	Self-disclosure	0.13***	0.26***	0.13***	−0.15***	−0.14***	−0.14***	–	0.30***	0.34***
8	Disclosure perpetration	0.13***	0.22***	0.14***	−0.13***	−0.12***	−0.12***	0.53***	–	0.69***
9	Disclosure victimization	0.14***	0.24***	0.13***	−0.11***	−0.12***	−0.13***	0.39***	0.67***	–

*p < 0.05, **p < 0.01, and ***p < 0.001.

### 3.3 Regression analysis on cyberbullying from disclosure of private information and perceived school climate

Regression analyses were conducted to further explore the impact of disclosure of private information and perceived school climate on cyberbullying. The results are summarized in Table 3. Among the school climate factors, student support in the school environment had a negative impact on cyberbullying perpetration (*B* = −0.34, *p* < 0.001), victimization (*B* = −0.34, *p* < 0.001), and dual roles in perpetration-victimization (*B* = −0.35, *p* < 0.001). This suggests that higher levels of student support were associated with lower levels of cyberbullying experiences across all three types. Conversely, opportunities for autonomy had a positive impact on cyberbullying perpetration (*B* = 0.08, *p* < 0.05), victimization (*B* = 0.09, *p* < 0.05), and dual roles in perpetration-victimization (*B* = 0.09, *p* < 0.05). This indicates that higher levels of opportunities for autonomy were associated with higher levels of cyberbullying experiences across all three types. No significant relationship was observed between teacher support and cyberbullying in either school settings, suggesting that teacher support did not have a significant impact on the occurrence of cyberbullying.

**Table 3:** Regression analysis on cyberbullying from disclosure of private information and perceived school climate.

Parameter	Standardized B		
	Cyber perpetration	Cyber victimization	Cyber Perpetration-victimization
Teacher support	0.03	0.01	0.03
Opportunities for autonomy	0.08*	0.09*	0.09*
Student support	−0.34***	−0.34***	−0.35***
Self-disclosure	0.02	0.18***	0.02
Disclosure perpetration	0.08*	0.05*	0.09**
Disclosure victimization	0.07*	0.09**	0.06*
*R* ^2^	0.13	0.20	0.14
*F*	44.18	71.17	45.96
*p*	<0.001	<0.001	<0.001

*p < 0.05, **p < 0.01, and ***p < 0.001. Gender and age were controlled in the analysis.

In terms of the disclosure of private information, self-disclosure in cyberspace had a positive impact on cyberbullying victimization (*B* = 0.18, *p* < 0.001). This implies that individuals who engage in self-disclosure of private information online are more likely to experience cyberbullying victimization. Furthermore, disclosure perpetration and victimization were positively related to cyberbullying experiences, with *B* values ranging from 0.05 to 0.09 (*p* < 0.001). This suggests that individuals who engage in the perpetrating or being victims of disclosure of private information are more likely to experience cyberbullying. Student support exhibited a protective effect, while opportunities for autonomy increased the risk of cyberbullying.

### 3.4 Simple slope analysis of school types between student support and cyberbullying

**Figure 1:**
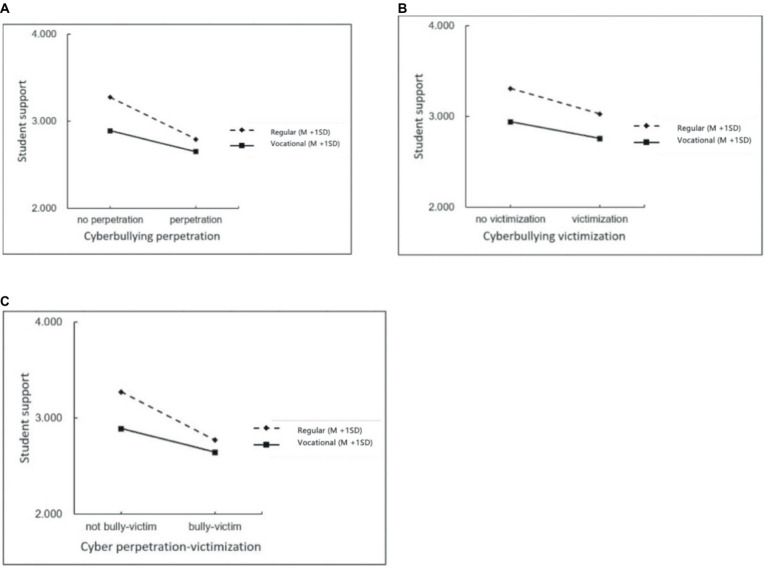
Simple slope analysis of school types between student support and cyberbullying. **(A)** Student support and cyber perpetration (*p* < 0.001). **(B)** Student support and cyber victimization (*p* < 0.001). **(C)** Student support and cyber perpetration-victimization (*p* < 0.001).

A simple slope analysis was conducted to further explore the relationship between student support and cyberbullying, as shown in Figure 1. The results revealed that student support was negatively related to cyberbullying perpetration and victimization. Importantly, the effect of student support on cyberbullying perpetration, victimization, and perpetration-victimization was stronger in regular schools than in vocational schools (all *p* < 0.001). This finding suggests a more pronounced association between student support and cyberbullying in regular school settings. The simple slope analysis provides additional insights, highlighting the variable impact of student support across different school types.

## 4 Discussion

### 4.1 Comparisons between students in regular and vocational schools

This study examined the relationship between school climate, the disclosure of private information, and cyberbullying among Chinese adolescents. It found that 35.6% of the participants had experienced cyberbullying at some point. Notably, students from vocational school reported a higher prevalence of cyberbullying compared to those from regular schools. Additionally, they scored lower in their perception of school climate and were less likely to disclose private information than their counterparts in regular schools. These findings align with recent research on cyberbullying in Chinese vocational schools (42). The disparity in perceived school climate, which includes elements such as teacher support, student support, and school autonomy, may stem from the higher status of teachers and more frequent interactions with classmates in regular schools (14). In these environments, students often assist teachers with class management tasks, such as monitoring behavior and organizing meetings. Conversely, vocational school students may feel excluded and marginalized, potentially leading to feelings of uncertainty, disengagement, poor teacher–student relationships, peer victimization, and increased dropout risks (43). The higher incidence of cyberbullying among vocational school students may prompt them to engage in riskier online activities, including less cautious disclosure of private information, as they cope with feelings of anger and fear (44). This creates a vicious cycle that practitioners need to understand. It is important for practitioners to recognize that adolescents, particularly those in vocational schools who suffer higher levels of victimization, may be more reticent about disclosing private information. Understanding these dynamics is crucial for practitioners and policymakers to tackle the unique challenges these adolescents face. Developing effective interventions requires fostering a positive school climate, promoting safe disclosure practices, and mitigate the risk of cyberbullying.

### 4.2 Prevention of privacy disclosure

Participants in the study commonly disclosed personal information, including birthdays, names, personal photos and videos, and academic grades. However, the prevalence of both perpetrating disclosure and falling victim to it was found to be much lower than that of self-disclosure. This aligns with previous research, which suggest that while cyberspace offers opportunities for virtual interactions, many individuals still rely on their offline networks, and the benefits of these interactions are mutually beneficial (45). Several factors influence the tendency to disclose information online, including perceived risk, age, cost-benefit appraisal, self-efficacy, and the type of social networking site used. For instance, older adolescents are more cautious about online information disclosure (46). Platforms like Instagram attract young users who value the control they offer over privacy, followers, and the impressions made on their audience. Educators and parents play a crucial role in raising awareness about the risks associated with online privacy, particularly regarding the disclosure of private information on social networking sites (45). It is feasible and important to proactively educate adolescents about making conscientious choices and fostering positive online interactions. By awareness promotion and guidance, educators and parents can empower adolescents to navigate the online world responsibly, make informed decisions about information disclosure, and develop healthy and respectful online interactions.

### 4.3 Promoting positive school climate

The results of our study revealed negative relationship between perceived student support and both cyberbullying perpetration and victimization. Furthermore, this effect was more pronounced among students in regular schools. These findings align with previous research, which has indicated that a decrease in perceived student support can contribute to increased aggression and cyberbullying among adolescents (14). Trusted teacher–student relationships often lead to more frequent conversations and enhanced mutual trust (43). However, our study did not identify a significant relationship between teacher support and cyberbullying, which could be attributed to the stronger influence of peer interactions. Peer interactions can provide a sense of closeness and support in challenging situations (47), potentially overshadowing the role of teacher support. Additionally, cyberbullying is a complex issue influenced by various factors beyond the school environment, including social media use and individual motivations. Within vocational school settings, student responses to teacher support differed, likely reflecting the unique contexts these schools provide. Some vocational school students may be more hesitant to report cyberbullying to teachers, perceiving them as inefficient or ineffective in addressing such issues. A sense of feeling unsafe at school and mistrust towards teachers has been associated with psychosocial difficulties, bullying victimization, and cyberbullying perpetration. Perpetrators may seek social support in both school and cyberspace to release negative emotions and engage in maladaptive behaviors (47). These findings highlight the significant responsibility of schools in preventing cyberbullying. Improving student support is essential for safeguarding adolescents both inside and outside of school, including in online environments (14).

### 4.4 Navigating cyberbullying prevention

Online self-disclosure and disclosure victimization were found to have a positive relationship with cyberbullying victimization among adolescents, which is consistent with the majority of previous studies examining the link between disclosure of private information and cyberbullying (48). Social networking sites encourage users to create personal profiles, which promotes self-disclosure as a means of developing relationships. This can lead to a more relaxed attitude towards privacy boundaries. However, posting private information online carries various risks, including identity theft and cyberbullying victimization. Young adolescents, in particular, often fail to recognize the seriousness of privacy breaches while overestimating the social rewards of sharing private information (25). This misperception is partly due to the belief that privacy violations are an inherent part of the functionality of many social networking sites. Given these findings, it is highly recommended to implement school-based cyberbullying prevention programs that address the risks associated with online self-disclosure behaviors. These programs should also emphasize training on coping strategies to students who have been victims of disclosure. By raising awareness about online privacy risks and equipping students with the skills to navigate these challenges, educators can help mitigate the negative consequences of cyberbullying and promote a safer online environment for adolescents.

### 4.5 Limitations

This study has several limitations that should be considered when interpreting its findings. First, the cross-sectional design of this study precludes the establishment of causal relationships between variables. Longitudinal studies are recommended to better understand the dynamics between school climate and cyberbullying, as they can provide more robust evidence. Second, to accurately determine attribution, it is important to control for experiences of offline victimization, such as school bullying. Future research should include measures of offline victimization to gain a comprehensive understanding of the relationship between school climate and cyberbullying. Third, the reliance on self-reported questionnaires to assess the disclosure of private information and experiences of cyberbullying perpetration may result in underreporting due to social desirability bias. Future studies could employ alternative methods, such as data collection from teachers or website administrators, or the use of behavioral experiments to supplement self-report measures. This would provide a more comprehensive understanding of cyberbullying dynamics (47). Additionally, the study sample was limited to students from regular and vocational schools in two specific cities in China, which may limit the generalizability of the findings to wider national or international context. Future studies should strive to include more diverse samples to investigate regional and demographic variations, including differences in social skills, socialization processes, and peer influences.

### 4.6 Implications

Despite the limitations mentioned, the findings of this study have significant implications. It was observed that school climate is negatively associated with cyberbullying and the disclosure of private information in both regular and vocational schools. Policies underscore the necessity of enhancing child protection schemes in cyberspace, yet evidence on how to effectively construct these schemes is limited (47). This highlights the importance of considering offline factors to ensure online safety among adolescents. Given that adolescents spend a substantial portion of their time in school, it is crucial for educational institutions to create a youth-friendly campus environment that promotes positive development and provides a safe space for students (19). The rapid advancement of information technology and evolving patterns of online social interactions necessitate continuous research efforts in the field of privacy protection. Measures such as screening for keywords on websites can help mitigate the risks associated with disclosing private information. Policymakers should take note of these cost-effective approaches and consider the potential benefits of incorporating digital health education to promote child protection across diverse populations. Based on the findings of this study, it is recommended that school staff prioritize fostering teacher–student support, strengthening student alliances, and providing opportunities for autonomy to enhance the safety of schools for adolescents. Future research should delve deeper into the contextual factors that influence adolescents’ perceptions of school climate and explore the mechanisms underlying the relationships described above. Previous studies have also indicated that technologically proficient adolescents may be more effective in assisting their peers with technology-related issues (19). However, it is important to acknowledge that digital literacy levels may vary across regions with different income inequalities. In this regard, schools can offer platforms for cyberbullying victims to engage in discussions with trusted peers and seek support, thereby reducing associated distress (49). Interventions targeting the disclosure of private information can involve parents through school-based programs to provide timely support to potential victims. Bridging the digital divide across districts is also crucial to establish a supportive and universally applicable infrastructure for online child protection.

## Data availability statement

The raw data supporting the conclusions of this article will be made available by the authors, without undue reservation.

## Ethics statement

The studies involving humans were approved by The Hong Kong Polytechnic University. The studies were conducted in accordance with the local legislation and institutional requirements. Written informed consent for participation in this study was provided by the participants’ legal guardians/next of kin.

## Author contributions

QC: Conceptualization, Data curation, Formal analysis, Funding acquisition, Methodology, Project administration, Resources, Writing – original draft. JT: Writing – original draft. YZ: Writing – review & editing. KC: Supervision, Writing – review & editing.
